# Dynamic ultrasound imaging—A multivariate approach for the analysis and comparison of time-dependent musculoskeletal movements

**DOI:** 10.1186/1471-2342-12-29

**Published:** 2012-09-27

**Authors:** Tommy Löfstedt, Olof Ahnlund, Michael Peolsson, Johan Trygg

**Affiliations:** 1 Computational Life Science Cluster (CLiC), Department of Chemistry, Umeå University, Umeå, Sweden

**Keywords:** Ultrasound, Medical imaging, Wavelet transform, Musculoskeletal movements, Multivariate data analysis, O2PLS, Speckle tracking

## Abstract

**Background:**

Muscle functions are generally assumed to affect a wide variety of conditions and activities, including pain, ischemic and neurological disorders, exercise and injury. It is therefore very desirable to obtain more information on musculoskeletal contributions to and activity during clinical processes such as the treatment of muscle injuries, post-surgery evaluations, and the monitoring of progressive degeneration in neuromuscular disorders.

The spatial image resolution achievable with ultrasound systems has improved tremendously in the last few years and it is nowadays possible to study skeletal muscles in real-time during activity. However, ultrasound imaging has an inherent problem that makes it difficult to compare different measurement series or image sequences from two or more subjects. Due to physiological differences between different subjects, the ultrasound sequences will be visually different – partly because of variation in probe placement and partly because of the difficulty of perfectly reproducing any given movement.

**Methods:**

Ultrasound images of the biceps and calf of a single subject were transformed to achieve congruence and then efficiently compressed and stacked to facilitate analysis using a multivariate method known as O2PLS. O2PLS identifies related and unrelated variation in and between two sets of data such that different phases of the studied movements can be analysed. The methodology was used to study the dynamics of the Achilles tendon and the calf and also the Biceps brachii and upper arm. The movements of these parts of the body are both of interest in clinical orthopaedic research.

**Results:**

This study extends the novel method of multivariate analysis of congruent images (MACI) to facilitate comparisons between two series of ultrasound images. This increases its potential range of medical applications and its utility for detecting, visualising and quantifying the dynamics and functions of skeletal muscle.

**Conclusions:**

The most important results of this study are that MACI with O2PLS is able to consistently extract meaningful variability from pairs of ultrasound sequences. The MACI method with O2PLS is a powerful tool with great potential for visualising and comparing dynamics between movements. It has many potential clinical applications in the study of muscle injuries, post-surgery evaluations and evaluations of rehabilitation, and the assessment of athletic training interventions.

## Background

Medical imaging is a rich source of information in the diagnostic process. Variation in characteristics of interest can be identified, compared, and correlated to specific symptoms and clinical findings by analysing patterns observed within the imaged tissues. These patterns can be used to determine whether tissues are benign or malign, and intact or repaired. Imaging can thus be used to test the effectiveness of remedies and evaluate the effects of treatments for specific diseases.

The aim of our studies is to develop tools to detect, visualise and quantify skeletal muscle dynamics and functionality using ultrasound imaging and multivariate image analysis. The strategic significance of such tools is very high since they could be applied in muscle rehabilitation programmes (including sports medicine) and the treatment of neurological disorders (e.g. whiplash and fibromyalgia) and pain-related conditions (e.g. back pain).

The quality of ultrasound imaging has evolved rapidly, and modern equipment is capable of recording images with good spatial resolution at high frame rates. This makes ultrasound imaging suitable for the analysis of both structural and functional aspects of muscles. For example, it has been used to study tendon injuries in sports medicine [1] and inflammation processes [2,3].

Ultrasound imaging has been used to study tissue movements for a couple of decades. A period of training is required before good registrations can be captured, but studies focusing on ultrasound validation of the tendon tracking have accurately quantified tendon displacements without reference to anatomical landmarks [4], and exhibit high repeatability within and between both subjects and examiners [5,6].

There are important differences between structural imaging studies and dynamic studies of functional movements. In the first case, the patient remains still while the clinician moves the ultrasound probe. Conversely, in dynamic studies, the patient is asked to perform a movement and the clinician holds the ultrasound probe still relative to an anatomical landmark [7,8]. The latter approach was adopted in this work. Furthermore, structural assessments are based on single images while dynamic analyses are based on series of images recorded while the patient performs specific movements. Dynamic and structural studies thus have different and complementary emphases.

Ultrasound images of tissue consist of a set of intensity values that form a mosaic image in shades of grey. This mosaic consists of speckles that create patterns, which can be regarded as “fingerprints” of specific tissue components. If the probe remains in the same position during a movement, the changes in the speckle patterns represent movements in the tissue. These movements can be directly and actively followed using speckle-tracking software. A Region of Interest (ROI) is an area that is identified in the first frame of an ultrasound sequence and then followed frame by frame. Movements of the tissue over the course of an image sequence can be “observed” and quantified by monitoring changes in the position of the ROI. If the discrete wavelet transform is used, these changes in intensity, which represent actual muscle tissue movements, are indirectly and passively observed as they pass by the “observer” (the variable, the wavelet coefficient). When images of this kind are analyzed using techniques such as O2PLS, movements of specific muscle tissues are represented by multiple variables that together describe individual phenomena within the sequence. The data analysis aspect is therefore to identify the systematic structures in the pixel variations present in the subsequent images that best describe the movement being performed.

Ultrasound imaging has an inherent problem when comparing two different measurement series or comparing image sequences from two or more subjects. Since different subjects will invariably have various physiological differences, there will be visual differences between their ultrasound sequences. This is partly due to differences in probe placement and partly to the difficulty of exactly duplicating any given movement.

This paper describes a method that facilitates the comparison of two ultrasound sequences based on combining the O2PLS method and Multivariate Analysis of Congruent Images (MACI). MACI [9,10] is a powerful tool for analysing and visualising the dynamics of muscle tissues in ultrasonic B-mode grey scan image sequences [10]. It is based on the discrete wavelet transform that effectively compresses image sequences while preserving information relating to time and shape.

This article explains the flexibility of MACI and demonstrates that it can be readily used to compare ultrasound image sequences. The method has two components: the discrete wavelet transform and multivariate data analysis using O2PLS. The wavelet transform is used to extract position, size and shape information that is present in greyscale B-scan ultrasound image sequences captured while performing muscular movements. This approach has two key advantages over conventional image analysis [9]. First, while conventional methods deal only with the intensities of individual pixels, MACI also analyses pixel intensities across larger regions as a whole. This eliminates problems with non-congruency. Second, the application of O2PLS to the wavelet-transformed images makes it possible to compare sequences and identify similarities and differences. In previous studies, we have performed MACI using PCA [10] rather than O2PLS. O2PLS is advantageous in this context because it can be used to compare two full length registrations, e.g. one from a healthy individual and another from an injured person, or registrations from a person before and after an intervention. Third, the results of multivariate O2PLS analyses can be further manipulated using a range of statistical tools to perform comparisons, cluster analyses, discrimination analyses, and so on. All of these increase the scope for describing the functional and dynamic aspects of skeletal muscle movements.

## Methods

### Modelling Tissue Dynamics

The basis of image analysis is that an *M x N* two-dimensional digital intensity image can be regarded as a function, *I(x, y)*, with an intensity value for every point *(x, y)* of the image [11].

When several such 2D images are stacked upon one-another, a multivariate image space is created. In this work, stacks of ultrasound images were created in which each layer of the stack represented a specific time point. In such a multivariate image space, when looking from the top of the stack, each location (i.e. pixel) is represented by a series of greyscale values (with one value for each image in the sequence) and so the stack contains a very large amount of highly correlated data. Various unfolding procedures can be used to analyse these 3D data structures [12-14].

The feature extraction procedure used in this work is based on the discrete 2D wavelet transform (2D-DWT). In this method, images are compressed by choosing the wavelet coefficients that hold the most information about the ultrasound sequence. These coefficients are found by ordering them according to their variance (importance) and choosing the *n* variables with the highest variance, i.e. the coefficients with the highest information content. The selected wavelet coefficients are then stored in a regular two-way data table where each row represents an image and each column contains the wavelet coefficients for the images. Multivariate analysis is then performed on the two-way data tables of wavelet coefficients.

### O2PLS

O2PLS is an extension of Partial least squares regression (PLS-R) and Orthogonal projections to latent structures (OPLS) [15] invented by Johan Trygg in 2002 [16,17]. O2PLS creates a model linking two matrices *X* and *Y*. In this article, *X* and *Y* are two ultrasound sequences that could represent two movements performed by the same subject or the same movements performed by two subjects. In O2PLS, the variation in *X* and *Y* is divided into four parts. The first two describe the joint X↔Y covariation, i.e. what in *X* is linearly related to *Y*, and what in *Y* is linearly related to *X*. The remaining parts describe what in *X* is unrelated to *Y* and what in *Y* is unrelated to*X*. All of these parts can be analysed separately. O2PLS is very similar to PLS regression [18-20] and OPLS, and has properties in common with both methods, but O2PLS increases the *interpretability* of the resulting model.

The O2PLS model comprises the four model matrices and two residual matrices. The relationships between these matrices are as follows:

$$X=T_{k}P_{k}^{T}+T_{o,l}P_{o,l}^{T}+E$$

and

$$Y=U_{k}C_{k}^{T}+U_{o,m}Q_{o,m}^{T}+F$$

where the *o* subscript refers to the unrelated variation in *X* and *Y*, *k*, *l* and *m* are the number of latent variables in each matrix, *T* contains the *X*-scores, *P* contains the *X*-loadings, *U* contains the *Y*-scores, and *Q* contains the *Y*-loadings. *E* and *F* are the residual matrices.

O2PLS was performed using SIMCA-P+ 12.0 (MKS Umetrics AB, Umeå, Sweden). The cross validation procedure in SIMCA-P+ 12.0 was used to determine how many related and unrelated components should be used. Note that the number of components needed for any given analysis depends on the purpose of the study. The number of components suggested by cross validation is equal to the number of dimensions in the space that are significantly different from noise. Conversely, it is usually sufficient to consider only the two “largest” dimensions (components) to identify the movement phases (as discussed below). When analysing other important forms of variation, however, it may be necessary to consider greater numbers of components in order to extract the most important information.

### Multivariate analysis of congruent images

Multivariate analysis of congruent images (MACI) is used to find and express patterns over multivariate image spaces in order to classify or study relationships between images [9]. The main goals of multivariate image analysis are first to compress the highly correlated data in terms of a few linear combinations of the intensity values, and second to preserve the spatial information in the images.

A set of images is said to be congruent if they have been properly pre-processed (i.e. transformed) such that each element in any given image corresponds to the same element in all of the other images. If the image series is not fully congruent to begin with (as is generally true), it must be made so [4,12-14,21]. In this work, the discrete 2D wavelet transform (2D-DWT) using the Symlet 8 wavelet basis was applied to each image in the series to make them congruent. The 2D-DWT was used to extract spatial (position) and frequency (shape and size) data from the images.

Once obtained, the wavelet coefficients for each image (i.e. observation) are recorded in the rows of an ordinary two-way data table that can be analysed using conventional multivariate methods such as PCA or O2PLS to extract information. In this work, these data sets were analysed using MACI in conjunction with O2PLS.

The development of wavelet-based texture analysis methods has greatly expanded the scope of multivariate data analysis techniques of this kind in recent years [22].

**Figure 1:**
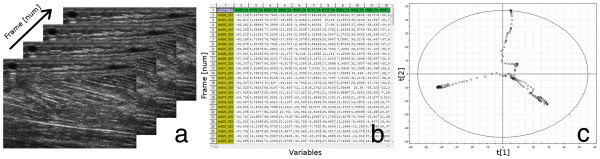
**The schematic principle of MACI.** The principle of transforming an ultrasound image sequence (**a**) into a score plot (**c**) by means of the wavelet transform (**b**). (**a**) A multivariate image space made up of a series of images from an ultrasound loop. (**b**) The congruent images are stored as the rows of an ordinary two-way data table. This means that each row corresponds to a point, or frame, in time; the columns contain the wavelet coefficients. (**c**) A two-component score plot of the table of wavelet coefficients in which the second score vector is plotted against the first score vector. Each circle in this plot represents one image or one time point in the ultrasound sequence.

The principles of MACI are illustrated in Figure 1, which shows the transformation of a series of B-scan ultrasound images into a score plot using the 2D-DWT.

### Speckle tracking

The acoustic patterns in an ultrasound signal change in response to the activation of the muscle being scanned when the probe is held in a fixed position relative to an anatomical landmark. These acoustic markers, or speckle patterns, remain relatively stable over time and can therefore be followed frame-by-frame in a sequence of images [23].

In the examples discussed below, speckle tracking was performed using an in-house post-processing software package in order to obtain a reference analysis of some of the different phases of the movements and to show how much movement there is in different anatomical regions at different times.

The first step in speckle tracking is to specify a rectangular region of interest (ROI) in a particular frame. The objective is then to find the region in the next frame that is most similar to the selected region according to some criterion. The objective is thus to find the values of Δx and Δy that minimise

$$\varepsilon =\sum_{y}\sum_{x}{\left[I\left(x,y,t\right)-I\left(x+\Delta x,y+\Delta y,t+1\right)\right]}^{2}w\left(x,y\right)\text{,}$$

Where *I* is the image intensity, *x* and *y* are the pixel coordinates at time (or for frame) *t*, and *w* is a weighting function that takes a value of 1 in the simplest case.

In the examples presented below, the ROIs were defined in the first frame of each series and the movements were captured in the following frames using a frame-by-frame approach. There are two directional components to the movements of the ankle: towards and away from the knee (dorsal and plantar flexion, respectively). Vector flow fields were created from the speckle tracks in order to illustrate changes in direction and to highlight the movement patterns of the different anatomical regions in each phase.

The algorithm does not always follow speckles correctly when the analysed tissue changes rapidly or is significantly deformed. This can also happen if out-of-plane motions occur. However, current research indicates that despite these limitations, speckle tracking is well suited for studying skeletal muscle movements [24,25].

The speckle tracking algorithm used in the in-house speckle tracking software is the recent optical flow method developed by Farnebäck [26], as implemented in the open source computer vision library (OpenCV) version 2.0 (http://opencv.willowgarage.com/wiki/).

The accuracy and reproducibility of results obtained using our in-house speckle tracking software has been tested, yielding good to excellent intra-class correlation coefficients (unpublished data).

#### Experimental setup

Two examples are presented to illustrate the usefulness of O2PLS MACI. The first example illustrates its reliability and deals with captures of the Achilles tendon in a single subject performing a specific ankle movement. The second example concerns two very disjunctive captures (one longitudinal and one transversal) of the biceps brachii of the same subject, performing a maximal voluntary contraction.

When capturing the ultrasound loops, the ultrasound system (Vivid 7, GE Healthcare, Horten, Norway) was used in conjunction with a linear 12 MHz ultrasound probe. Movements were captured at 78.6 frames per second (FPS) with a resulting time resolution of approximately 13 ms between frames; the lateral resolution was 0.5 mm.

Since only the captures of the first example were controlled, the captures of the second example had to be synced during post-processing. This was done by specifying a beginning and an end frame for the movement, and linearly removing frames located between these two points within the longer sequence.

### Example 1: Capturing the movement of the Achilles tendon

**Figure 2:**
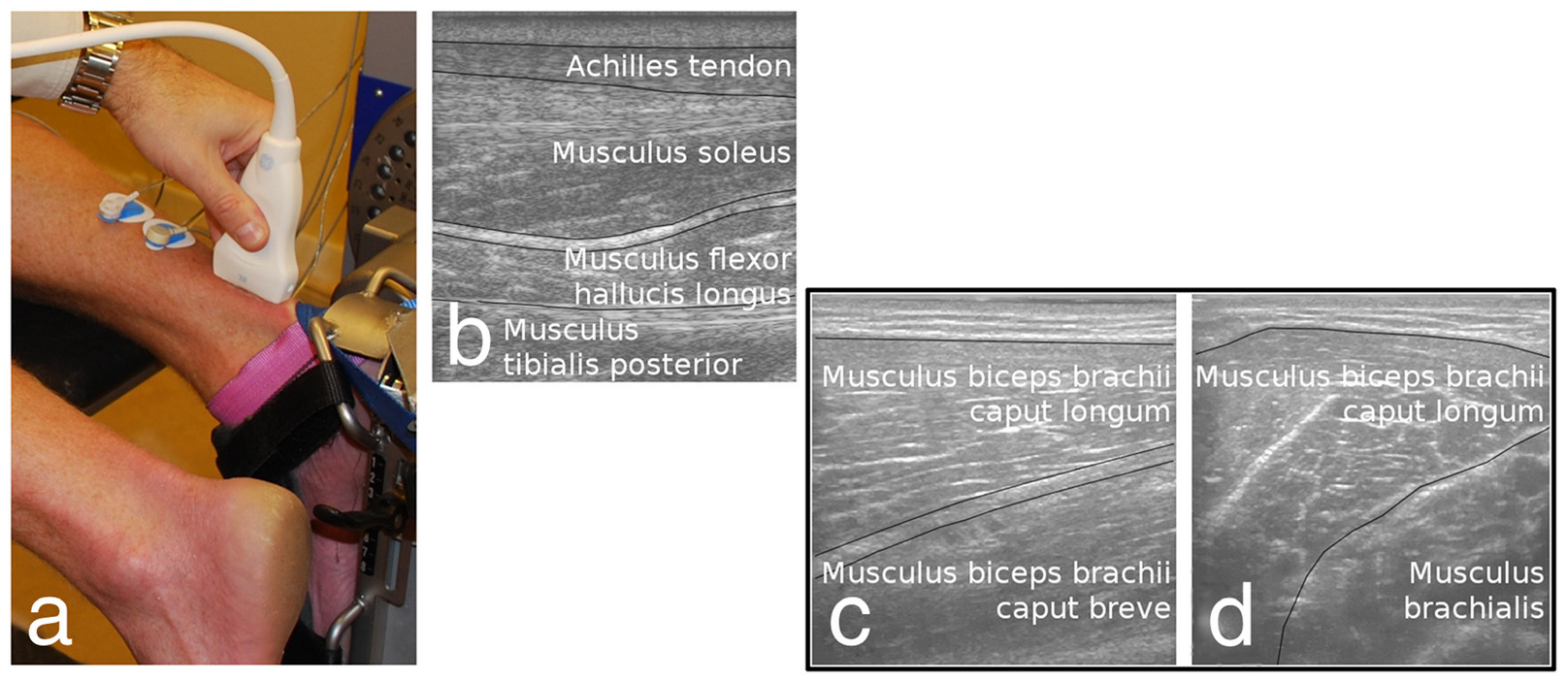
**Experimental setup and the resulting ultrasound sequences.** (**a**) The passive movement (Example 1) was performed lying face down with the foot strapped to an isokinetic dynamometer. The ultrasound probe was then held in the researcher’s hand, just above the subject’s malleolus. (**b**) The anatomy of the calf viewed from the probe surface towards the interior of the calf. The marked anatomic areas are the areas discussed in Example 1. Figures (**c**) and (**d**) show the anatomical regions in the biceps considered in Example 2. (**c**) is the longitudinal capture and (**d**) is the transversal capture.

The first example deals with an unloaded passive dorsal flexion ankle movement and illustrates the reliability of the O2PLS-MACI method, demonstrating that O2PLS extracts the same information from two separate sequences showing the same healthy subject performing a single movement. The movement was performed over a period of ~7.3 s with the ankle being moved from 20 degrees plantar to 15 degrees dorsal flexion. This movement was repeated twice in each sequence. The hand-held probe was positioned longitudinally over the posterior portion of the Achilles tendon, 3 cm proximal to the lateral malleolus. The anatomical regions that were captured are shown in Figure 2b.

### Example 2: Longitudinal and transversal captures

The second example demonstrates that the method facilitates comparisons between two orthogonal ultrasound registrations, in this case a longitudinal and transversal projection of the same muscle (the biceps brachii). Two ultrasound sequences of the biceps brachii were captured during a 20% maximal voluntary contraction (MVC) calculated from an MVC (isometric contraction). The ultrasound probe was placed in two perpendicular orientations on the first and second runs. For the first run, it was held in a longitudinal direction such that the ultrasound sequence captured the muscles along the muscle fibres. For the second, the probe was rotated along its own axis such that it was oriented in a transverse direction and the resulting ultrasound sequence captured the intersection of the muscle fibres. The two registrations thus have only a small area in common corresponding to the thickness of the probe. The arm was fixed at an angle of 120 degrees angle (where 180 degrees corresponds to full flexion of the arm) in a KinCom dynamometer. The maximal voluntary contraction (MVC) was determined and the movement was performed at 20% of the MVC for ~3.6 s.

As one might expect, these two ultrasound sequences are visually very different, as seen in Figure 2c and Figure 2d. However, despite their visual differences, the registrations are assumed to contain enough common information about the actual underlying movement to enable the extraction and analysis of the similarities and differences in the movements.

### Ethical consideration

The research was conducted with informed consent, and has been exempted from formal ethical approval by the Regional Ethical Review Board.

## Results

The results show that the O2PLS models for these experiments do very well at describing the relationships between sequences of registrations. O2PLS can thus be used not only for similar sequences of the same muscles, but also for sequences captured in different ways. In all cases, it was possible to identify and correlate the different phases of the movements in both sequences.

### Example 1: Captures of the Achilles tendon

When MACI was first applied to ultrasound sequences, it was used in conjunction with PCA [10]. While PCA can be used to construct models for each sequence, it cannot readily be used to study the relationships between the resulting models. When O2PLS is used instead to model the relationship between the sequences instead, one obtains a clear picture of the correlations between the sequences that can be studied in detail.

The O2PLS model that was obtained had 24 predictive (i.e. shared) components that described 73% of the variance in the first capture and 64% of the variance in the second, with no orthogonal components. This means that a significant amount of shared variation was found, and neither sequence contained any information that was distinguishable from noise and not present in the other.

**Figure 3:**
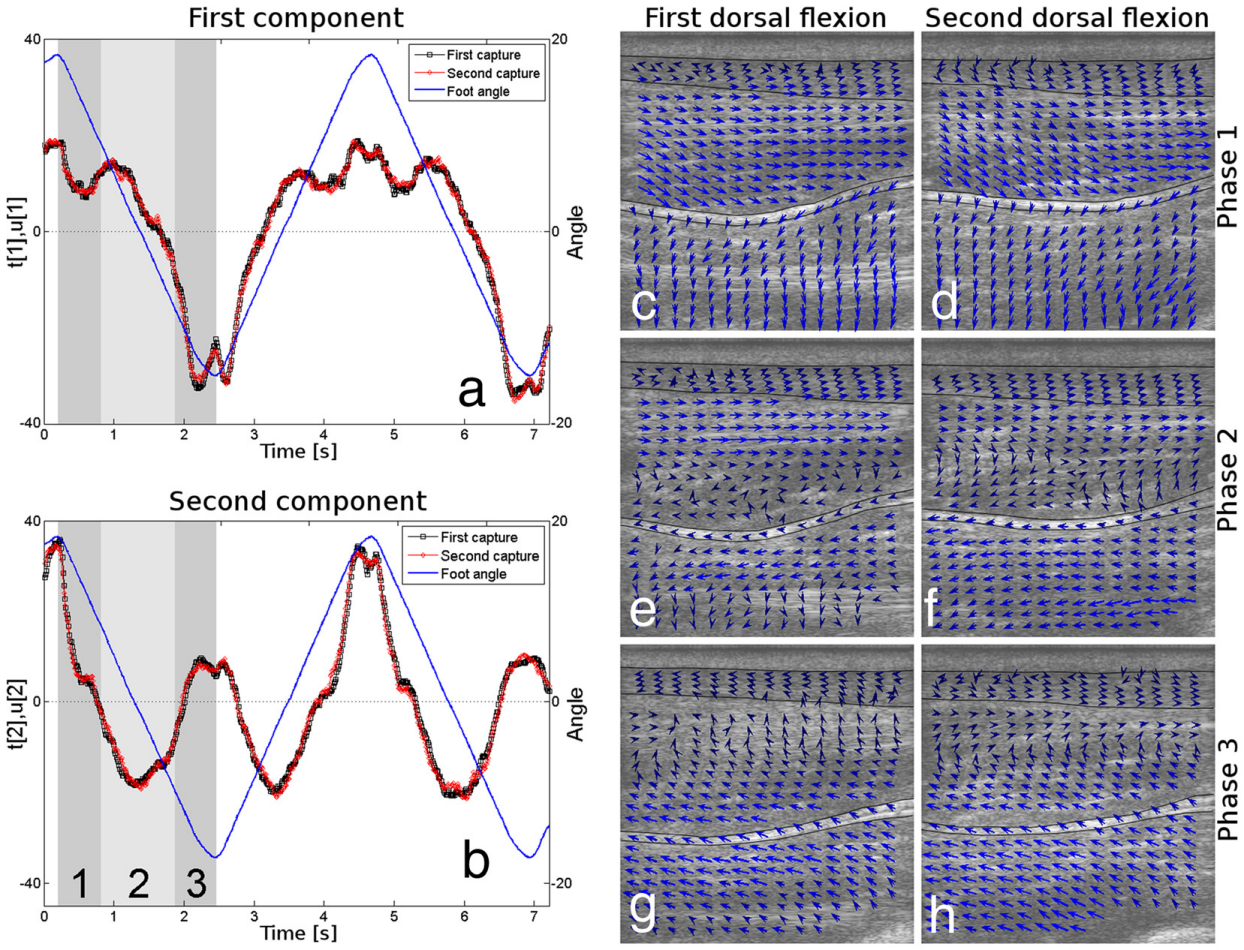
**Example 1: Reliability study focusing on the Achilles tendon.** The first (**a**) and second (**b**) score vectors are plotted together with the average angle measured by the isokinetic dynamometer for the two movements. Both the plantar and the dorsal flexions are clearly captured by both score vectors. The dorsal flexion is divided into three phases: Phase 1 is the end of the plantar flexion, Phase 2 is the transition from plantar to dorsal flexion and Phase 3 is the beginning of the dorsal flexion. The phases are marked in both Figure (**a**) and (**b**); in the case of Figure (**b**), the different phases are indicated by the numbers 1, 2 and 3. The corresponding speckle tracking vector field plots are shown in (**c**) and (**d**) for Phase 1, (**e**) and (**f**) for Phase 2 and (**g**) and (**h**) for Phase 3. The directions of the vectors indicate the direction of motion of the corresponding tissues.

See Figure 3 for a visual correlation of the frames during the ankle movement. The first and second O2PLS components are shown in Figures 3a and b, respectively, together with the goniometric data for the ankle as it moves between the directions of flexion. The turning phases of the movement were thus easily identified.

Four movement phases are clearly visible when looking at the ultrasound sequences. These are the plantar flexion turning point, the dorsal flexion, the dorsal flexion turning point, and the plantar flexion. The first three of these phases are highlighted in Figures 3a and b as Phase 1, 2 and 3. Speckle tracking vector flow fields [27,28] were created for each of these phases in order to illustrate how the phases differ. These vector fields are shown in Figures 3c-h for the first and the second captures. Figures 3c and d correspond to Phase 1 (the plantar flexion turning point), Figures 3e and f to Phase 2 (the dorsal flexion), and Figures 3g and h to Phase 3 (the dorsal flexion turning point). Four calf regions were analysed when these vector fields were created (see Figure 2), namely the Achilles tendon, Musculus soleus, Musculus flexor hallucis longus, and Musculus tibialis posterior, along with the fascia between the Soleus and Hallucis muscles. The outlines of these regions are indicated in Figures 3c-h (the Musculus flexor hallucis longus and Musculus tibialis posterior were analysed together). The arrows of the vector field represent the movement within each area, with the length of each arrow corresponding to the speed and length of the movement in its vicinity. Note that the arrow lengths are comparable within each image but cannot be used to make comparisons between the different images in Figure 3c-h.

Note in Figures 3a-b how well the components correlate with respect to both the first and second components. Actually, all 24 of the predictive components in T and U have correlation coefficients exceeding 0.94. This means that the ultrasound sequences are very similar and can be modelled together very well.

Overall, this example demonstrates that when two controlled repetitive movements were analysed using O2PLS, identical dynamic behaviour was observed in both sequences.

### Example 2: Longitudinal and transversal captures

The two sequences examined in this example are very different, but the purpose of the O2PLS method is to extract common information present in both sequences. In this case the relevant information concerns dynamic aspects of the movement being studied.

**Figure 4:**
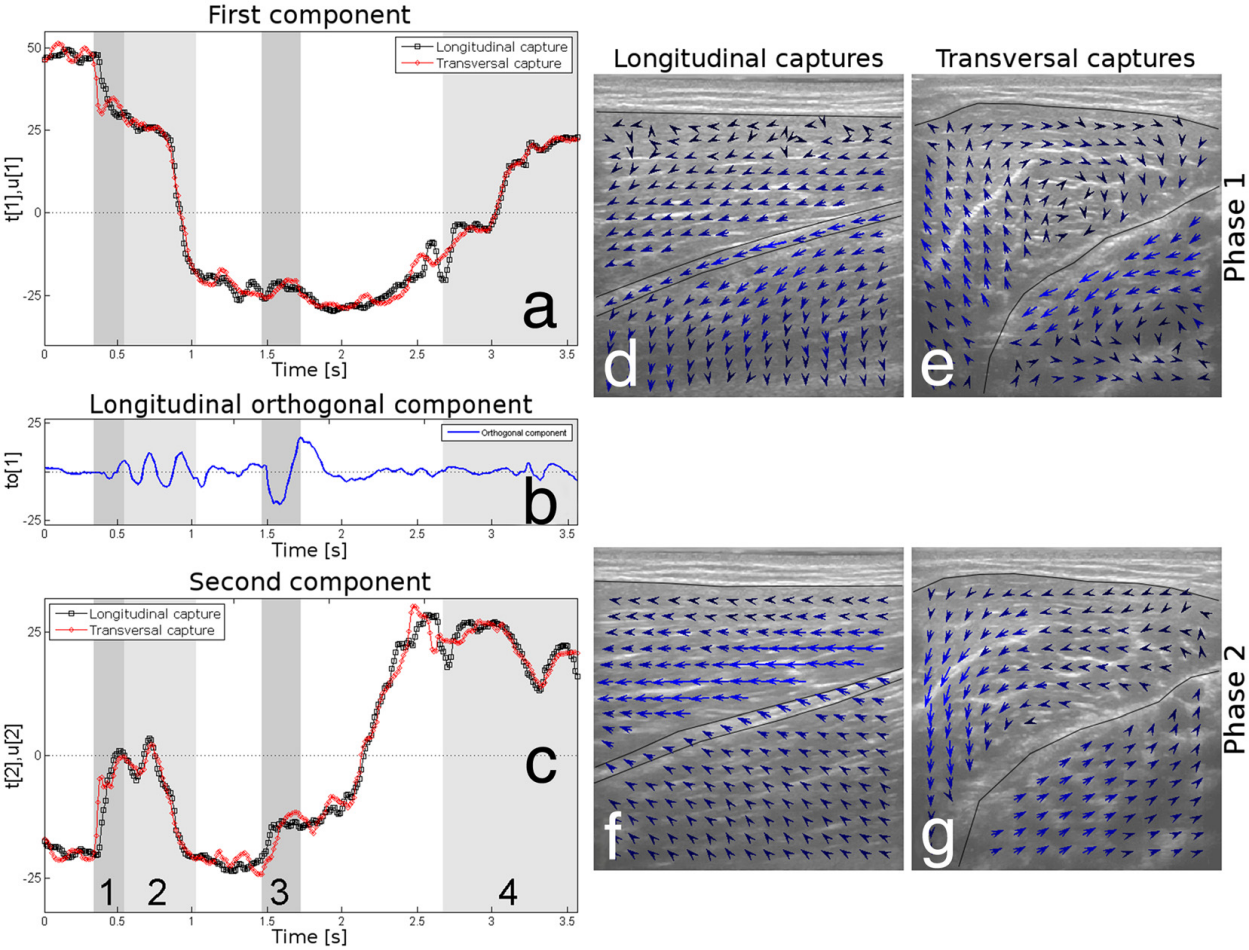
**Example 2: Longitudinal and transversal captures.** The first (**a**) and second (**c**) score vectors are plotted along with the orthogonal component (**b**). The movement has three phases: An active dynamic concentric phase (during muscle activation), an active static contraction phase (when the muscle is constantly tense) and a passive eccentric phase (when the arm returns to the baseline position). The active dynamic concentric phase has two sub-phases, Phase 1 and Phase 2. In Phase 1, it is mainly the Musculus biceps brachii caput breve and Musculus brachialis that are active (see Figures (**d**) and (**e**)); and in Phase 2 it is mainly the Musculus biceps brachii caput longum that is active (see Figures (**f**) and (**g**)). This is seen in Figure (**a**) as a plateau starting at the end of Phase 1 and at the beginning of Phase 2, and as a local maximum in Figure (**b**). In Phase 3 there is a small twitch in the longitudinal capture that is captured clearly in the orthogonal component, Figure (**b**). Phase 4 is the end of the movement, the passive eccentric phase when the muscle returns to rest.

The O2PLS analyses of these sequences did indeed capture a common underlying dynamic behaviour. As can be seen in Figures 4a and c, there is a similar and congruous pattern in both sequences. The O2PLS model that was found had 18 predictive components (describing 87% of the variance in the longitudinal and 80% of the variance in the transversal capture) and one component in the longitudinal capture that was orthogonal to the transversal capture. This means that a significant amount of shared variation was found, and that something of significance happened in the longitudinal capture that did not exist in the transversal capture.

The movement as performed had three phases: a contraction phase (during muscle activation), a static contraction phase (when the muscle was constantly tensed) and a relaxation phase (when the arm returned to its start position). These three phases are very well described by this O2PLS model, and especially in the first component. The contraction phase is actually composed of two sub-phases, which are indicated in Figures 4a-c and are referred to as Phase 1 and Phase 2. Phase 1 lasts from ~0.3 s to ~0.6 s and Phase 2 lasts from ~0.6 s to ~1.0s. The former mainly involves the short head of the Biceps brachii while the latter mainly involves the long head. This can also be seen in the speckle tracking vector field plots shown in Figures 4d and f, which correspond to Phase 1 and Phase 2, respectively.

The static contraction phase lasts from ~1.0 s to ~2.7 s and the dynamic eccentric phase lasts from ~2.7 s to the end of the sequence at ~3.6 s. The relaxation phase is shown as Phase 4 in Figures 4a-c.

The orthogonal component in Figure 4b suggests that something happened in the longitudinal capture during the static contraction phase, in the area marked as Phase 3 in Figures 4a-c. Specifically, a twitch in Phase 3 in both heads of the Biceps brachii is apparent in the longitudinal ultrasound sequence. Since this twitch did not occur in the transversal capture, the behaviour was captured in the orthogonal component, $t_{o,1}$, of the longitudinal capture. This orthogonal component also had high values during the contraction phase and at the end of the relaxation phase, indicating that differences occurred there as well.

The second component, Figure 4c, suggests that there is dynamic activity in the static contraction phase as well; this is probably due to the difficulty of maintaining a constant force during the MVC (this was also mentioned in reference [10] as well and thus once again confirms previous findings [29]).

One interesting thing to notice is that even though the muscle is relaxed at the start and the end of the movement, the score plots shown in Figures 4a and c do not begin and end at the same position with the same score value. This is probably because of a slight difference in tension in the muscles before and after the movement.

A drawback of this experiment is the lack of EMG data. However, the force curve obtained from the KinCom gave information regarding the length of the activation phase capturing the submaximal phase of the MVC (20%) movement.

## Discussion

The main conclusion from these studies is that the combination of O2PLS with MACI represents a very useful and important extension to the MACI method. When used in conjunction with O2PLS, MACI can accurately identify and explain the common variation found in two ultrasound sequences. MACI modelling with PCA, as described in [10], provides a method for effectively compressing and extracting dynamic behaviour in a single video sequence. The O2PLS version of this method described herein facilitates comparisons between two blocks of data (two video sequences). O2PLS extracts common variation and allows for the analysis of discrepancies and non-related variation between the two blocks.

It is very useful to be able to compare different functional movements when using ultrasound image analysis to study musculoskeletal processes. When using the MACI method with PCA, only one ultrasound sequence could be described at a time. Conversely, when using O2PLS it is possible to analyse two full-length registrations simultaneously. This greatly increases the scope of the method, making it possible to compare registrations from a healthy and an injured person, different phases of a specific movement, or registrations from a person before and after an intervention. As such, the method clearly has considerable potential for use in medical applications.

The O2PLS method is an inductive, data-driven approach that extracts the information that is the most important for describing a particular sequence. It is thus the method rather than the analyst that decides which parts of the image should be examined. This unbiased data exploration technique therefore has great potential in clinical situations.

The O2PLS MACI method will thus add value to medical image analysis in different ways. Traditional image analysis methods focus on extracting specific features from single sequences. Conversely, O2PLS MACI extracts all of the information contained in every image pertaining to the movement in question and can be used to analyse them simultaneously in a comparative fashion. It could potentially be used to analyse the dynamics within individual muscles and tendons. Alternatively, image fields showing several muscles could be analysed in order to describe activity patterns.

It was possible to separate the studied functional movements into distinct phases that correspond to actual directions of motion as verified by visual inspection. The vector flow fields generated by the analyses illustrate the movements occurring within each phase in more detail, providing information that is extremely useful in a medical context because it can be used to compare the effects of different movements and determine how a specific intervention improves a given situation or to identify correlations between interventions and subjective symptoms or clinical findings.

The second case study examined in this work suggests that the angle of the ultrasound probe does not greatly affect the results obtained. This is encouraging, because it means that it would not be necessary to ensure that the probe was positioned in exactly the same way each time when capturing ultrasound sequences in the clinic. That is to say, the underlying movements can be compared even when sequences are captured from different angles or in slightly different ways.

Some variation is inevitable in all ultrasound registrations. Functional movements are also in and of themselves hard to repeat exactly, even among athletes who strive for years to achieve high levels of consistency when performing specific movements. It is therefore interesting to consider the variation between two repetitions of individual movements, both in terms of the common factors and the differences between them. As such, it is important to use standardized protocols that facilitate such comparisons whenever possible.

This study was methodological rather than clinical. The results presented suggest that the new method has considerable potential in the clinic but more testing and development will be required before it can be used in clinical applications, and the limitations of the case studies presented should be considered in this context.

## Conclusions

MACI with O2PLS is able to consistently extract meaningful variability from ultrasound sequences. The O2PLS MACI method is thus applicable to studies of intra-individual relationships in ultrasound sequences of musculoskeletal tissues.

All of the studied sequences were modelled well, i.e. there were strong correlations between the O2PLS score vectors and the overlap between the sequences was substantial. Any variation present in both analysed sequences can thus be extracted efficiently, indicating that the shared variation corresponds to dynamic behaviours that exist in both movements.

When the shared variation was studied, the patterns that arose in the score vectors corresponded to changes in the nature of the movement (i.e. transitions from one phase to another) that could be verified by visual inspection. This indicates that MACI with O2PLS can identify different phases of functional movements.

Analyses of repeated movements generated similar score patterns, implying that the method exhibits good repeatability.

MACI with O2PLS is thus a powerful tool for studying variations and relationships between images in ultrasound image sequences with a broad range of potential applications. One such potential application concerns studies on the functionality healthy and injured muscles or the effects of training. It could also be useful for studying neuro-motor related diseases such as multiple sclerosis. The potential applications of the method will be explored in more detail in future studies from our laboratories.

To our knowledge, there were previously no methods for inductively studying whole image sequences. The development of MACI with O2PLS has now made it possible to do this.

## Competing interests

TL received financial support from MKS Umetrics AB, Umeå, Sweden.

## Authors' contributions

TL performed the data analysis and wrote the manuscript together with OA and MP. MP generated the data. JT and MP designed and coordinated the study. All of the authors read and approved the final manuscript.

## Pre-publication history

The pre-publication history for this paper can be accessed here:

http://www.biomedcentral.com/1471-2342/12/29/prepub
